# Supplementary material for: The Prevalence of Tick-Borne Encephalitis Virus in Wild Rodents Captured in Tick-Borne Encephalitis Foci in Highly Endemic Lithuania
Source: Viruses. 2024 Mar 13;16(3):444. doi: 10.3390/v16030444 (PMC10974453; doi:10.3390/v16030444)
Supplement: Supplementary file 1 [file viruses-16-00444-s001.zip › viruses-2887328-supplementary.pdf]

Supplementary material:

Simkute E. et al. The Prevalence of Tick-Borne Encephalitis Virus in Wild Rodents Captured in Tick-Borne Encephalitis Foci in Highly Endemic Lithuania

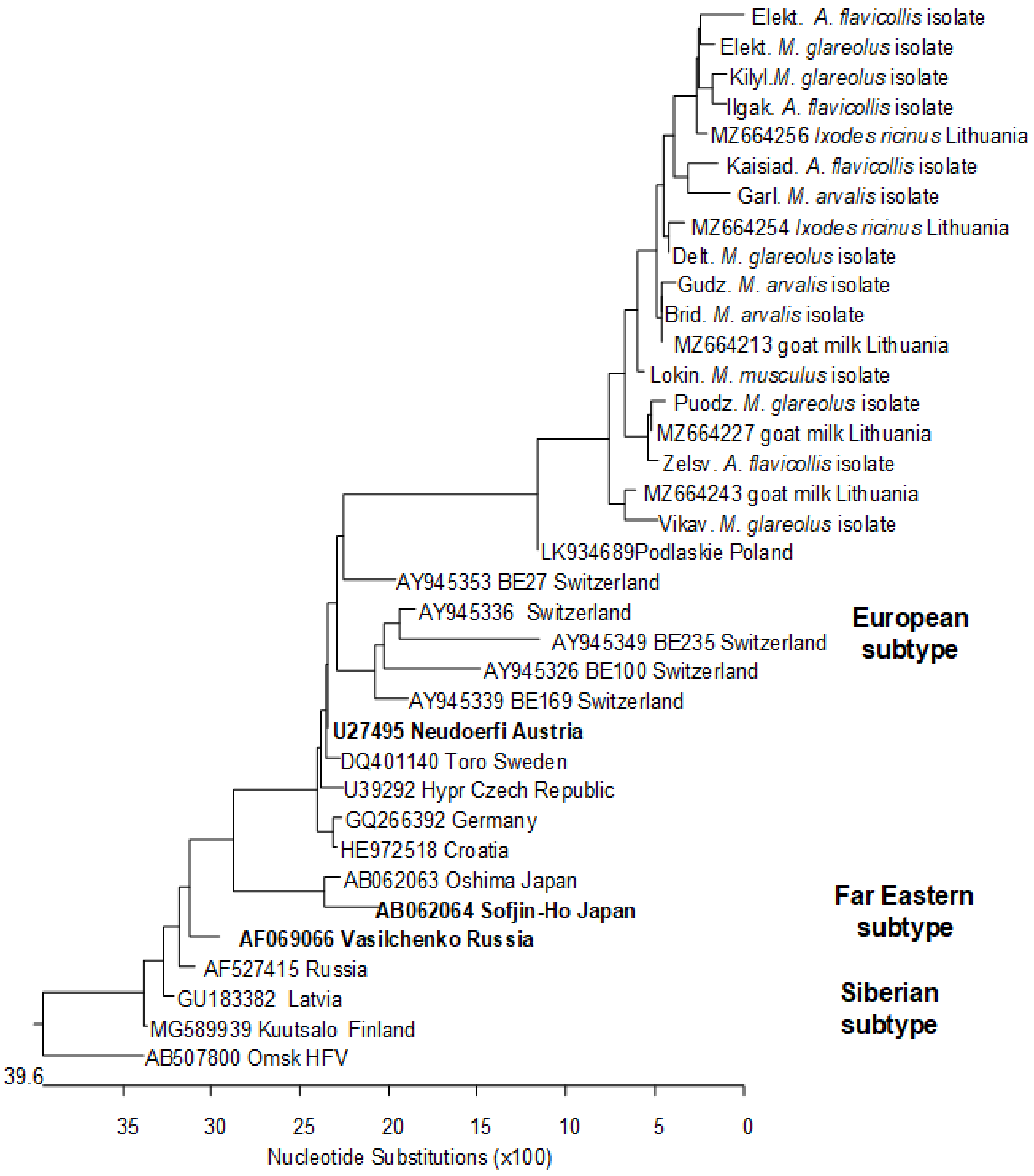

Figure. S1. Phylogenetic tree of the obtained TBEV isolate sequences based on NCR genome fragment. The Omsk hemorrhagic fever virus was used as an outgroup. Sequences of different TBEV strains and closely related flaviviruses chosen from the NCBI GenBank database were used for phylogenetic comparisons. Multiple alignment of all sequences was created using ClustalW software (Clustal, Dublin, Ireland) in MEGA X package. The neighbor-joining method was used for phylogenetic tree construction with 100 bootstrapping replicates.

Table S1. Results of TBEV RNA prevalence rate in rodent brain and internal organ mix sample isolates which were PCR-negative in suspensions (n = 30), grouped according to the month of rodent trapping.

Date of rodent trapping	Number of trapped rodents (total/TBEV RNA-positive in suspensions/TBEV RNA-positive in isolates)	TBEV RNA prevalence rate (%) in suspension samples	TBEV RNA prevalence rate (%) in rodent sample isolates which were PCR-negative in suspensions	Average monthly air temperature (°C)	Rodent trapping sites and TBEV RNA prevalence rate (%) in suspension samples
**March 2019**	34/28/33	82.4	15.2 (CI 95 % 5.1 – 31.9)[1]	3 (1 – 3.7)	Elektrenai (84.6); Garliava (72.7); Grabuciskes (100); Ilgakiemis (90)
**April 2019**	8/5/8	62.5	37.5 (CI 95 % 8.5 – 75.5)[2]	8.6 (5 – 15.1)	Bridai (25); Narepai (100)
**October 2019**	24/15/22	62.5	31.8 (CI 95 % 13.9 – 54.9)	9.3 (6 – 13.2)	Kaisiadorys (42.9); Naujasodis (60); Pakalniskes (50); Zelsva (87.5)
**November 2019**	23/15/21	65.2	28.6 (CI 95 % 11.3 – 52.2)	4.9 (- 1.2 – 8.3)	Kazlu Ruda (62.5); Krokialaukis (66.7)
**January 2020**	31/28/31	90.3	9.7 (CI 95 % 2.0 – 25.8)[2]	2.8 (0.6 – 5.1)	Gudziunai (100); Kivyliai (100); Puodziai (70); Elektrenai (100)
**March 2020**	9/9/9	100	0 (CI 95% 0 – 33.6)*	3.3 (0.5 – 5.5)	Deltuva (100); Lokine (100)
**May 2020**	10/4/10	40	60 (CI 95 % 26.2 – 87.8)	10.2 (7.2 – 13.1)	Lekeciai (25); Vilkaviskis (50)

Note: [1] Including rodent which was PCR-positive only in fetal sample suspension. [2] Including rodent which brain sample was not taken. [*]One-sided 97.5% confidence interval.

Table S2. Results of TBEV RNA prevalence rate in brain and internal organ mix suspension and isolate samples of different rodent species.

Name of the rodent trapping location	Latitude coordinate	Longitude coordinate	Date of rodent trapping	Number and species of trapped rodents (total/TBEV RNA positive in suspensions/TBEV RNA positive in isolates)	TBEV RNA prevalence rate in suspension samples (%)	TBEV RNA prevale nce rate in isolate samples (%)
**Gudziunai**	56.0888	23.2805	Jan 2020	*A. flavicollis* 2/2/2<br>*C. glareolus* 1/1/1<br>*M. arvalis* 9/9/9	100	100
**Bridai**	56.0226	23.3263	Apr 2019	*A. flavicollis* 2/0/2	25	100

				M. arvalis 2/1/2		
**Deltuva**	55.2857	24.6144	Mar 2020	*C. glaroelus* 1/1/1	100	100
				M. musculus 1/1/1		
**Elektrenai**[1]	54.7763	24.6290	Mar 2019	*A. flavicollis* 8/6/8	84.6	100
				C. glareolus 1/1/1		
				M. arvalis 1/1/1		
			Jan 2020	*A. flavicollis* 2/2/2	100	100
				C. glareolus 1/1/1		
**Garliava**	54.8011	23.8756	Mar 2019	*A. flavicollis* 8/7/8*	72.7	100
				C. glareolus 2/1/2		
				M. arvalis 1/1/1		
**Grabuciskes**	54.8947	24.1574	Mar 2019	*M. arvalis* 2/2/2	100	100
**Ilgakiemis**	54.7860	23.8677	Mar 2019	*A. flavicollis* 4/3/3	90	90
				C. glareolus 2/2/2		
				M. arvalis 3/3/3		
				M. musculus 1/1/1		
**Kaisiadorys**	54.8511	24.4561	Oct 2019	*A. flavicollis* 5/2/3	42.9	71.4
				M. musculus 1/0/1		
				A. agrarius 1/1/1		
**Kazlu Ruda**	54.7371	23.4609	Nov 2019	*A. flavicollis* 5/4/5	62.5	100
				C. glareolus 3/1/3		
**Kivyliai**	56.3566	22.7115	Jan 2020	*C. glareolus* 5/5/5	100	100
				M. arvalis 1/1/1		
**Krokialaukis**	54.4277	23.4847	Nov 2019	*A. flavicollis* 12/7/10	66.7	86.7
				C. glareolus 1/1/1		
				A. sylvaticus 2/2/2		
**Lekeciai**	54.5213	23.0375	May 2020	*A. flavicollis* 2/0/2	25	100
				M. musculus 2/1/2		
**Lokine**	54.3275	24.4900	Mar 2020	*A. flavicollis* 4/4/4	100	100
				M. musculus 3/3/3		
**Narepai**	54.9491	24.0171	Apr 2019	*A. flavicollis* 1/1/1	100	100
				C. glareolus 3/3/3*		
**Naujasodis**	54.9517	23.9873	Oct 2019	*A. flavicollis* 5/3/5	60	100
**Pakalniskes**	54.7371	24.6999	Oct 2019	*A. flavicollis* 2/0/2	50	100
				C. glareolus 1/1/1		
				M. arvalis 1/1/1		

**Puodziai**	54.3580	24.6600	Jan 2020	*A. flavicollis* 4/4/4	70	100
				C. glareolus 6/3/6		
**Vilkaviskis**	54.6294	23.1872	May 2020	*A. flavicollis* 1/0/1*	50	100
				C. glareolus 2/0/2*		
				M. arvalis 2/2/2*		
				M. musculus 1/1/1		
**Zelsva**	54.4070	23.4578	Oct 2019	*A. flavicollis* 8/7/8	87.5	100

Note: [1] Rodent samples were collected at different months in the same location. [*] A fetal sample was taken from rodents.
